# Injectable Hydrogel Loaded With Umbilical Cord Blood‐Derived Exosomes Promotes Periodontal Bone Regeneration via the TLR4/NF‐κB Signalling Pathway

**DOI:** 10.1111/jcmm.71347

**Published:** 2026-09-27

**Authors:** Dongao Zhu, Mingkun Liu, Yue Fan, Lv Lang, Xin Luo, Zhihong Zhang, Zuojun Shen, Honghong Liu

**Affiliations:** ^1^ Department of Stomatology, the First Affiliated Hospital of USTC, Division of Life Sciences and Medicine University of Science and Technology of China Hefei China; ^2^ College of Stomatology Bengbu Medical University Bengbu Anhui China; ^3^ Department of Laboratory Medicine, the First Affiliated Hospital of USTC, Division of Life Sciences and Medicine University of Science and Technology of China Hefei China

**Keywords:** bone regeneration, exosomes, hydrogel, NF‐κB, periodontitis, TLR4, umbilical cord blood

## Abstract

Periodontitis is an immune‐mediated inflammatory disease characterised by progressive alveolar bone loss, which can lead to tooth loss in severe cases. Existing therapeutic agents have limited efficacy in reversing the associated inflammatory bone defects. Exosomes (Exos), enriched with bioactive molecules, exhibit significant potential in anti‐inflammation and osteogenic regeneration. In this study, an injectable and photocurable composite hydrogel sustained‐release system (GA‐Exos) loaded with human umbilical cord blood‐derived exosomes (UCB‐Exos) was constructed. In vitro and in vivo experiments demonstrated that GA‐Exos possesses excellent performance, enabling sustained release of exosomes for approximately 21 days. It significantly promotes the proliferation and differentiation of osteoblasts, upregulates the expression of osteogenic markers (e.g., RUNX2, ALP, and OPN), reduces the levels of pro‐inflammatory cytokines (e.g., TNF‐α and IL‐1β), and inhibits inflammatory cell infiltration and osteoclast differentiation. mRNA sequencing and validation experiments confirmed that UCB‐Exos inhibits the activation of the TLR4/NF‐κB pathway by downregulating the expression of TLR4, LBP, and CD14, as well as the phosphorylation of P65. In conclusion, GA‐Exos enhances periodontal bone regeneration through the regulation of the TLR4/NF‐κB pathway, providing a novel and effective strategy for periodontitis treatment. This system is expected to break through the limitations of existing therapies and bring new insights for clinical applications.

## 1 Introduction

Periodontitis is a highly prevalent oral disease globally. Beyond inducing symptoms such as gingival swelling, bleeding, and tooth mobility, it is closely linked to systemic conditions, including cardiovascular diseases and diabetes mellitus [1, 2, 3]. Currently, the primary treatment involves mechanical debridement to remove dental plaque and tartar, supplemented by local and systemic antibiotic therapy [4, 5]. However, this approach has limitations such as incomplete debridement, increased risk of antibiotic resistance, and significant surgical trauma [6, 7]. Furthermore, it only exerts antibacterial effects but fails to effectively regulate the host immune response or promote periodontal bone tissue regeneration [8]. The treatment of progressive alveolar bone loss and defects associated with periodontitis remains a clinical challenge. In recent years, therapeutic strategies based on bioactive molecules have emerged as a research focus in the field of periodontal bone regeneration.

Exosomes (Exos) are a subpopulation of nanoscale extracellular vesicles (EVs) actively secreted by cells. They carry bioactive substances such as miRNAs, transcription factors, and proteins, playing crucial roles in intercellular communication, immune regulation, and tissue regeneration [9, 10]. Compared with traditional cell‐based therapy, exosomes possess advantages such as low immunogenicity, high stability, and ease of storage and transportation, thereby emerging as a novel cell‐free therapeutic strategy [11]. Moreover, exosomes have been proven to possess both tissue regeneration‐promoting and potent anti‐inflammatory properties. They can activate osteogenic signals and inhibit osteoclastic activity in inflammatory microenvironments, demonstrating great application potential in the field of bone tissue regeneration [12, 13, 14, 15].

Human umbilical cord blood (UCB) is obtained from healthy mothers who undergo rigorous prenatal screening. Its collection is non‐invasive with minimal ethical controversy. Exosomes derived from UCB (UCB‐Exos) exhibit higher concentration and activity compared to those from adult peripheral blood [16, 17, 18], and have shown promising therapeutic potential in wound healing [19], cell proliferation and migration [20], as well as bone regeneration and anti‐inflammation [21]. Notably, anti‐inflammatory and osteogenic activities are key to addressing alveolar bone defects associated with periodontitis. However, research on the application of UCB‐Exos in periodontitis treatment remains scarce. A key bottleneck limiting the clinical translation of exosomes is their delivery system: Conventional injection approaches typically result in rapid in vivo clearance, which hinders the maintenance of effective concentrations at the lesion site [22, 23]. Hydrogels can overcome this limitation by providing local protection and sustained release of exosomes. Among them, the composite photocrosslinkable hydrogel (GA) composed of gelatin methacryloyl (GelMA) and sodium alginate methacryloyl (AlgMA) balances cytocompatibility and mechanical stability, enabling prolonged action of bioactive substances [24, 25, 26]. Nevertheless, the efficacy of GA loaded with UCB‐Exos for periodontitis treatment remains unclear.

Against this background, this study aims to develop an injectable, UCB‐Exos‐loaded photocurable composite hydrogel (GA‐Exos) for the repair of inflammatory bone defects caused by periodontitis through the long‐term sustained release of exosomes. First, the physicochemical properties of GA‐Exos, the in vitro sustained release rate of UCB‐Exos, and biocompatibility will be systematically characterised. Subsequently, the effects of UCB‐Exos on promoting cell proliferation, migration, and osteogenic differentiation will be verified at the cellular level. A rat periodontitis model will then be established to evaluate the regenerative repair efficacy of GA‐Exos on inflammatory bone defects. Finally, mRNA sequencing will be used to elucidate the potential molecular mechanisms underlying the osteogenic promotion of exosomes. This study provides a novel strategy for the regenerative treatment of inflammatory bone defects in periodontitis, with the main technical route shown in Figure 1.

**FIGURE 1:**
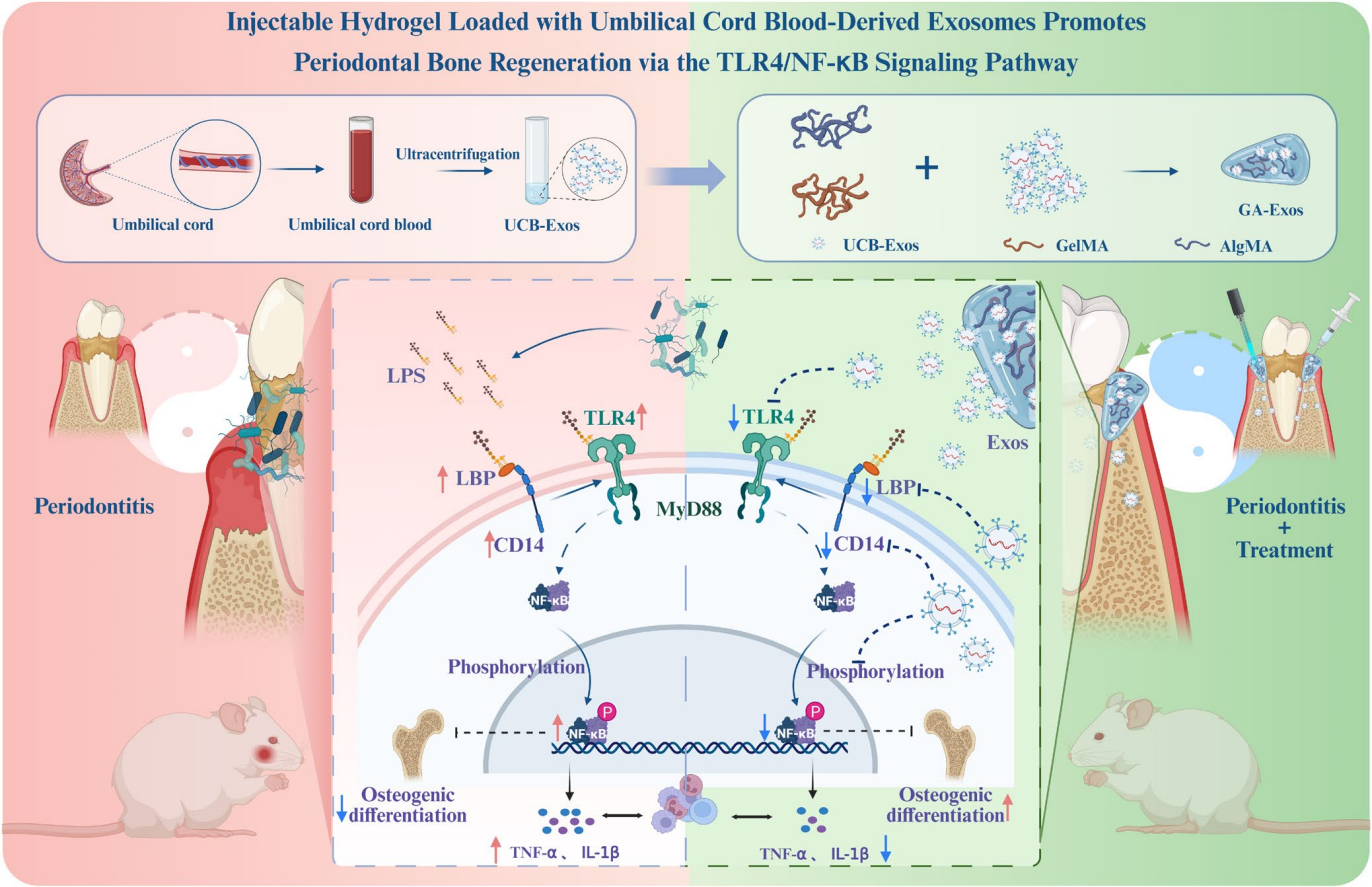
Graphical abstract (Created with BioRender.com).

## 2 Materials and Methods

### 2.1 Isolation and Characterisation of UCB‐Exos

This study was approved by the Medical Ethics Committee of the First Affiliated Hospital of the University of Science and Technology of China (approval number: 2024‐KY‐334). After obtaining informed consent from healthy pregnant women and their family members, umbilical cord blood was collected. Samples were stored at 4°C during transport and processed within 4 h post‐collection under sterile conditions. UCB‐Exos were isolated via ultracentrifugation, as follows: Briefly, umbilical cord blood samples were centrifuged at 1500 × g for 20 min at 4°C, followed by centrifugation at 3000 × g for 15 min at 4°C to harvest plasma. Cell debris was removed by centrifugation at 10,000 × g for 30 min at 4°C. The supernatant was filtered through a 0.22 μm filter membrane (Merck‐Millipore, Germany) and then centrifuged at 120,000 × g for 70 min at 4°C to pellet exosomes. The pellet was resuspended in 30 mL of pre‐cooled PBS and centrifuged again under the same conditions. Finally, the exosomes were resuspended in 200 μL of PBS. The protein concentration was determined using a BCA assay kit (Beyotime, China), and the samples were aliquoted and stored at −80°C. Fresh or single freeze–thaw aliquots were used to prevent vesicle aggregation.

First, the morphological characteristics of UCB‐Exos were observed using a transmission electron microscope (Hitachi, Japan). Then, Western blotting was performed to identify the specific markers of UCB‐Exos (CD63, TSG101, Alix) and the negative marker (Calnexin). Finally, the particle size distribution of UCB‐Exos was analysed using a NanoSight NS300 analyser (Malvern Instruments, UK).

### 2.2 Preparation of GelMA/AlgMA Composite Hydrogel

Gelatin methacryloyl (GelMA, New Material Xianjue, Suzhou) and sodium alginate methacryloyl (AlgMA, New Material Xianjue, Suzhou) solids were weighed and dissolved in PBS at a specific ratio. The mixture was sonicated in a 50°C water bath for 1 h until fully dissolved to prepare a prepolymer solution (Solution A) consisting of 16% (w/v) GelMA and 8% (w/v) AlgMA. Separately, lithium phenyl‐2,4,6‐trimethylbenzoylphosphinate (1 mg/mL, LAP, New Material Xianjue, Suzhou) was added to PBS containing 1 mg/mL UCB‐Exos (experimental group) or without UCB‐Exos (control group) and mixed thoroughly to obtain Solution B. Solution A and Solution B were vortexed at a volume ratio of 1:1, then photocrosslinked with 405 nm blue light for 30 s to solidify, yielding GA‐Exos (UCB‐Exos‐loaded) and GA (UCB‐Exos‐free) composite hydrogels, respectively.

### 2.3 Characterisation of Composite Hydrogels

#### 2.3.1 Scanning Electron Microscopy (SEM)

A MERLIN SEM (Carl Zeiss, Germany) was used for characterisation. Samples were freeze‐dried under vacuum at −80°C, sputter‐coated with Pt ions for 60 s to enhance conductivity, and then imaged. The collected images were imported into Nano Measurer software for statistical analysis of the microstructure.

#### 2.3.2 Fourier Transform Infrared Spectroscopy (FT‐IR)

Samples were ground with KBr at a mass ratio of 1:100 and pressed into pellets. A Bruker Tensor 27 FTIR spectrometer (Germany) was used to scan in the range of 400–4000 cm^−1^ to characterise the chemical properties of the GA composite hydrogel.

#### 2.3.3 Rheological Tests

Rheological measurements were performed using a HAAKE MARS III rheometer (Thermo Fisher, Germany) in oscillatory time‐sweep mode (strain = 1%, angular frequency = 5 rad·s^−1^). At 2 min into the experiment, 405 nm blue light (30 mW·cm^−2^) was irradiated for 30 s, and the evolution of storage modulus (G′) and loss modulus (G″) was recorded in real time.

#### 2.3.4 Compression Tests

A PY880‐B mechanical testing machine (Puyan, China) was used. Cylindrical samples (diameter = 8 mm, height = 8 mm) of 8% (w/v) GelMA and GA‐Exos photocurable hydrogels were prepared, equilibrated in 37°C PBS for 24 h, and blotted dry to remove surface moisture. The samples were compressed at a constant rate of 10 mm/min until failure, and the stress–strain curves were recorded in real time.

#### 2.3.5 Swelling Property Tests

Weighed and solidified composite hydrogel samples (W_o_) were immersed in 37°C PBS. At predetermined time points, samples were removed, blotted dry to remove surface liquid, and weighed (W_t_). Swelling equilibrium was defined as a mass change < 1%. The swelling ratio was calculated using the formula: (W_t_ − W_o_)/W_o_ × 100%.

#### 2.3.6 Degradation Property Tests

Freeze‐dried hydrogel samples were immersed in 37°C PBS. Samples were collected on days 1, 2, 3, 5, 7, 9, 11, 13, 15, 17, 19, and 21, freeze‐dried, and the remaining dry weight (W_t_) was measured. The degradation rate (DR) was calculated relative to the initial dry weight (W_o_) using the formula: DR (%) = (W_o_ − W_t_)/W_o_ × 100%.

#### 2.3.7 Exosome Sustained‐Release Property Tests

GA‐Exos composite hydrogel samples were immersed in PBS at a sample‐to‐solution volume ratio of 1:10 and incubated in a shaker at 37°C and 75 r/min. At 0, 1, 2, 3, 6, 9, 12, 15, 18, and 21 days, 200 μL of supernatant was collected, and an equal volume of PBS was added to maintain the total volume. According to the instructions of the BCA Protein Assay Kit (EpiZyme, Shanghai, China), the absorbance of the supernatant was measured to calculate the cumulative release rate of Exos.

### 2.4 Isolation and Culture of Cells

Rat bone marrow mesenchymal stem cells (BMSCs) were isolated from the femoral marrow of Sprague–Dawley (SD) rats via the whole bone marrow culture method. All animal experiments were conducted in accordance with the protocol approved by the Experimental Animal Ethics Committee of the First Affiliated Hospital of the University of Science and Technology of China (approval number: 2024‐N (A)‐108). Rats were anaesthetised by intraperitoneal injection of tribromoethanol (Lab Anim Tech, Beijing). Femurs and tibias were harvested, and the bone marrow cavity was exposed in a sterile laminar flow hood. The marrow cavity was rinsed 3 times with PBS, and the rinse solution was collected and filtered through a 40 μm cell strainer (Biosharp, Beijing). Cells were pelleted by centrifugation at 1000 × g for 5 min, resuspended in αMEM medium (Biosharp, Beijing) supplemented with 10% fetal bovine serum (FBS, Sigma‐Aldrich, USA), and seeded into culture dishes. The medium was changed every 3 days. When P0 generation cells reached 80% confluence, they were digested with 0.25% trypsin and passaged at a ratio of 1:2. P3 generation cells were used for subsequent experiments.

### 2.5 Cytotoxicity Assay of GA‐Exos Composite Hydrogel

Calcein/PI staining was used to detect cytotoxicity. After co‐culturing composite hydrogels with BMSCs for 48 h, cells were rinsed with PBS and incubated with Calcein/PI working solution (Beyotime, Shanghai) at 37°C in the dark. Images were captured using an inverted fluorescence microscope (Zeiss, Germany).

### 2.6 CCK‐8 Assay

A CCK‐8 kit (Beyotime, Shanghai) was used to evaluate cell proliferation. BMSCs were seeded into 96‐well plates at a density of 5 × 10^3^ cells/well. After 24 h, cells were co‐cultured with PBS, GA, or GA‐Exos, respectively. At 24 h and 48 h of co‐culture, CCK‐8 solution was added, and the plates were incubated at 37°C in the dark for 2 h. The absorbance at 450 nm was measured using a microplate reader (Thermo Fisher, USA).

### 2.7 Internalisation Assay

UCB‐Exos were labelled with PKH‐26 dye (Solarbio, Beijing), pelleted by ultracentrifugation, resuspended in αMEM, and sterilised through a 0.22 μm filter. Labelled UCB‐Exos were incubated with BMSCs for 12 h. Nuclei were stained with Hoechst 33,342 (Beyotime, Shanghai), and images were acquired using a laser scanning confocal microscope (Zeiss LSM800, Germany).

### 2.8 Cell Scratch Assay

BMSCs were seeded into 6‐well plates at a density of 5 × 10^5^ cells/well. When cells reached 90% confluence, a scratch was created at the bottom of the well using a 200 μL pipette tip. Cell debris was removed by rinsing with PBS, and cells were cultured in αMEM containing PBS or UCB‐Exos, respectively. Images were captured at 0 h, 24 h, and 36 h using an inverted microscope (Olympus, Japan), and the migration rate was analysed using ImageJ software.

### 2.9 Alkaline Phosphatase (ALP) and Alizarin Red S (ARS) Staining

BMSCs were seeded into 12‐well plates at a density of 2 × 10^5^ cells/well. When cells reached 80% confluence, they were divided into three groups: GA‐Exos group, GA group, and control group, and cultured in osteogenic induction medium (Pricella, Wuhan). ALP staining was performed on days 3 and 7: Cells were fixed with 4% paraformaldehyde (PFA, Servicebio, Wuhan) for 30 min, incubated with ALP staining solution (Beyotime, Shanghai) at room temperature in the dark for 1 h, and observed under an inverted microscope (Olympus, Japan). ARS staining was performed on days 14 and 21: Cells were fixed with 4% PFA for 30 min, rinsed with deionised water, stained with Alizarin Red S (Beyotime, Shanghai) for 30 min, and observed under an inverted microscope.

### 2.10 Western Blot Analysis

Total proteins were extracted using NP40 lysis buffer containing protease inhibitors. After separation by SDS‐PAGE, proteins were transferred to nitrocellulose (NC) membranes. The membranes were blocked with 5% non‐fat milk in TBST, incubated with primary antibodies overnight at 4°C, and then with secondary antibodies for 1 h at 37°C. Protein bands were visualised using an ultra‐sensitive ECL chemiluminescence kit (Beyotime, Shanghai). Primary antibodies included: TSG101, ALIX, CD63 (Abcam, UK); Calnexin, GAPDH (Proteintech, USA); RUNX2, OPN (Abclonal, China); ALP (GeneTex, USA); TLR4, phosphorylated P65 (P‐P65) (Zenbio, China); and P65 (Affinity, USA).

### 2.11 Quantitative Reverse Transcription Polymerase Chain Reaction (qRT‐PCR)

Total RNA was extracted from BMSCs and gingival tissues using TRIzol reagent (Invitrogen, USA). The concentration and purity of RNA were determined using a NanoDrop 2000 spectrophotometer. Complementary DNA (cDNA) was synthesised with a reverse transcription kit (Servicebio, Wuhan). qRT‐PCR was performed using TB Green Premix Ex TaqTM (Takara, Japan) with GAPDH as the internal reference gene. The relative expression levels of osteogenic‐related genes (RUNX2, ALP, OPN), inflammatory factors (TNF‐α, IL‐1β), and key pathway genes (LBP, CD14, TLR4) were calculated using the 2‐ΔΔCT method. Primer sequences are listed in Table 1.

**TABLE 1:** List of primer sequences.

Gene	Forward primer (5′‐3′)	Reverse primer (5′‐3′)
RUNX2	GGAACCAAGAAGGCACAGACAG	TGTCTGCCTGGGATCTGTAATCT
ALP	TGGTACTCGGACAATGAGATGC	GCTCTTCCAAATGCTGATGAGGT
OPN	TCTGATGAACAGTATCCCGATGC	ACTTGACTCATGGCTGGTCTTCC
TNF‐α	CTTCTCATTCCTGCTCGTG	TTTGGGAACTTCTCCTCCT
IL‐1β	TTCATCTTTGAAGAAGAGCCC	CTGTCTAATGGGAACATCACAC
GAPDH	CTGGAGAAACCTGCCAAGTATG	GGTGGAAGAATGGGAGTTGCT
LBP	GACATGAACTTGGAGCTTCTTGG	CATTGAACATGCCGACTTTGG
CD14	CGGGTTCCTACTCAGATTCTATTCG	CGAGCCAGGTATCCGTTGTT
P65	CAGATACCACTAAGACGCACCC	CTCCAGGTCTCGCTTCTTCACA
TLR4	CCAGGTGTGAAATTGAGACAATTG	AAGCTGTCCAATATGGAAACCC

### 2.12 Establishment of a Rat Periodontitis Model

Rat periodontitis was induced by ligation combined with lipopolysaccharide (LPS) injection. Twenty 8‐week‐old male SPF‐grade SD rats (300 ± 50 g) were acclimated to conventional feeding conditions. Rats were anaesthetised by intraperitoneal injection of tribromoethanol (10 mL/kg, Lab Anim Tech, Beijing), and 4–0 silk sutures were ligated around the cervical margin of the first molars. Local injections of LPS (1 mg/ml, Biosharp, Beijing) were administered twice a week under strict sterile conditions. After 4 weeks, the ligatures were removed, and plaque and calculus around the cervical margin were scraped off. Rats were randomly divided into three groups: GA‐Exos group, GA group, and control group. GA‐Exos, GA, or PBS was injected into the periodontal pockets (four sites: Mesial buccal, distal buccal, mesial palatal, and distal palatal) once every two weeks until slight overflow was observed. The injected materials were crosslinked by irradiation with 405 nm blue light for 30 s. The gingival index (GI) was recorded blindly at 2 and 4 weeks after treatment (0 = no swelling; 1 = mild swelling without bleeding; 2 = bleeding on probing; 3 = spontaneous bleeding). Rats were euthanised after 4 weeks of treatment, and maxillary bone blocks were harvested.

### 2.13 Cone‐Beam Computed Tomography (CBCT) Analysis

At 4 weeks after induction and 2 weeks after treatment, CBCT scanning was performed using a CBCT system (Meyer, China) with a resolution of 0.2 mm, operating at 70 kV and 5 mA. DICOM files were imported into DCTViewer software (Meyer, China) to obtain two‐dimensional images, which were used to evaluate the efficacy of inflammation induction and treatment.

### 2.14 Micro‐Computed Tomography (Micro‐CT) Analysis

Maxillary bone blocks were fixed with 4% PFA for 48 h, then scanned using a high‐resolution micro‐CT system (Hiscan XM, China) with a resolution of 17 μm, operating at 60 kV and 134 μA. The alveolar bone around the first molar was selected as the region of interest (ROI) using Hiscan Analyser 2017 software. The following parameters were measured: Bone volume fraction (BV/TV), mean CT value, trabecular thickness (Tb. Th), alveolar bone crest‐cementoenamel junction distance (ABC‐CEJ), and trabecular number (Tb. N).

### 2.15 Histological Analysis

Maxillary bone blocks were decalcified in 10% EDTA for 4 weeks and sectioned into 4 μm slices. Haematoxylin and Eosin (H&E) staining and tartrate‐resistant acid phosphatase (TRAP) staining were performed. The density of inflammatory cells and osteoclasts was observed under a microscope (Zeiss, Germany). Osteoclasts were counted blindly in 5 randomly selected fields of view under a 40× objective lens, and the average value was used for statistical analysis.

### 2.16 mRNA Sequencing Analysis

mRNA sequencing and analysis were performed by Personalbio Technology Co. Ltd. (Wuhan, China). Briefly, polyA mRNA was enriched using Oligo (dT) magnetic beads, fragmented into 300 bp fragments, and then reverse‐transcribed to synthesise cDNA. PCR amplification was conducted to construct libraries, and library quality was assessed using an Agilent 2100 Bioanalyzer. Paired‐end (PE) sequencing was performed on an Illumina platform.

DESeq was used for differential analysis of gene expression, and the conditions for screening differentially expressed genes were fold change in expression |log2FoldChange| > 1, significance *p*‐value < 0.05. Then, the Pheatmap software package in the R language was used to perform bidirectional clustering analysis on the union of differentially expressed genes and samples of all comparison groups. Euclidean method was used to calculate the distance, and the hierarchical clustering longest distance method (Complete Linkage) was used for clustering. Finally, GO, KEGG, and GSEA were used to enrich the differentially expressed genes (DEGs).

### 2.17 Statistical Analysis

All experimental results are expressed as the mean ± standard deviation (SD) of at least three independent experiments and analysed using GraphPad Prism 9 software (San Diego, CA, USA). Student's *t*‐test was used for comparisons between two groups. One‐way analysis of variance (ANOVA) was applied for comparisons among multiple groups. Two‐way ANOVA was used to analyse differences between two or more groups at different time points. Bonferroni post hoc test was performed to control Type I errors in multiple comparison scenarios. Statistical significance was defined as follows: (*) *p* < 0.05, (**) *p* < 0.01, (***) *p* < 0.001, (****) *p* < 0.0001, and “ns” indicates no significance.

## 3 Results

### 3.1 Characterisation of UCB‐Exos

UCB‐Exos were isolated from umbilical cord blood by ultracentrifugation (Figure 2A). Transmission electron microscopy (TEM) micrographs demonstrated that UCB‐Exos displayed a typical discoid morphology (Figure 2B). Nanoparticle tracking analysis (NTA) showed that the particle size of UCB‐Exos was concentrated at 136.1 nm (Figure 2C). Furthermore, Western blotting results demonstrated that UCB‐Exos highly expressed the exosomal markers CD63, TSG101, and ALIX, while Calnexin (a negative marker) was barely detectable (Figure 2D), further confirming the identity of exosomes. According to the MISEV2023 guidelines [27], while these physical characteristics and protein profiles are highly consistent with those of classical “exosomes,” strictly defining them as such requires definitive evidence of their endosomal biogenesis pathway. Since our ultracentrifugation protocol separates vesicles based on size and density, it is more scientifically accurate to classify these nanoscale vesicles as “small extracellular vesicles (sEVs).” However, to maintain consistency with conventional literature in this specific applied field, the term “exosomes” is retained throughout the text. To investigate whether UCB‐Exos could be internalised by cells, PKH26‐labelled UCB‐Exos were co‐cultured with BMSCs. After 12 h, fluorescent images showed that UCB‐Exos (red fluorescence) were distributed around the cell nuclei (blue fluorescence) (Figure 2E).

**FIGURE 2:**
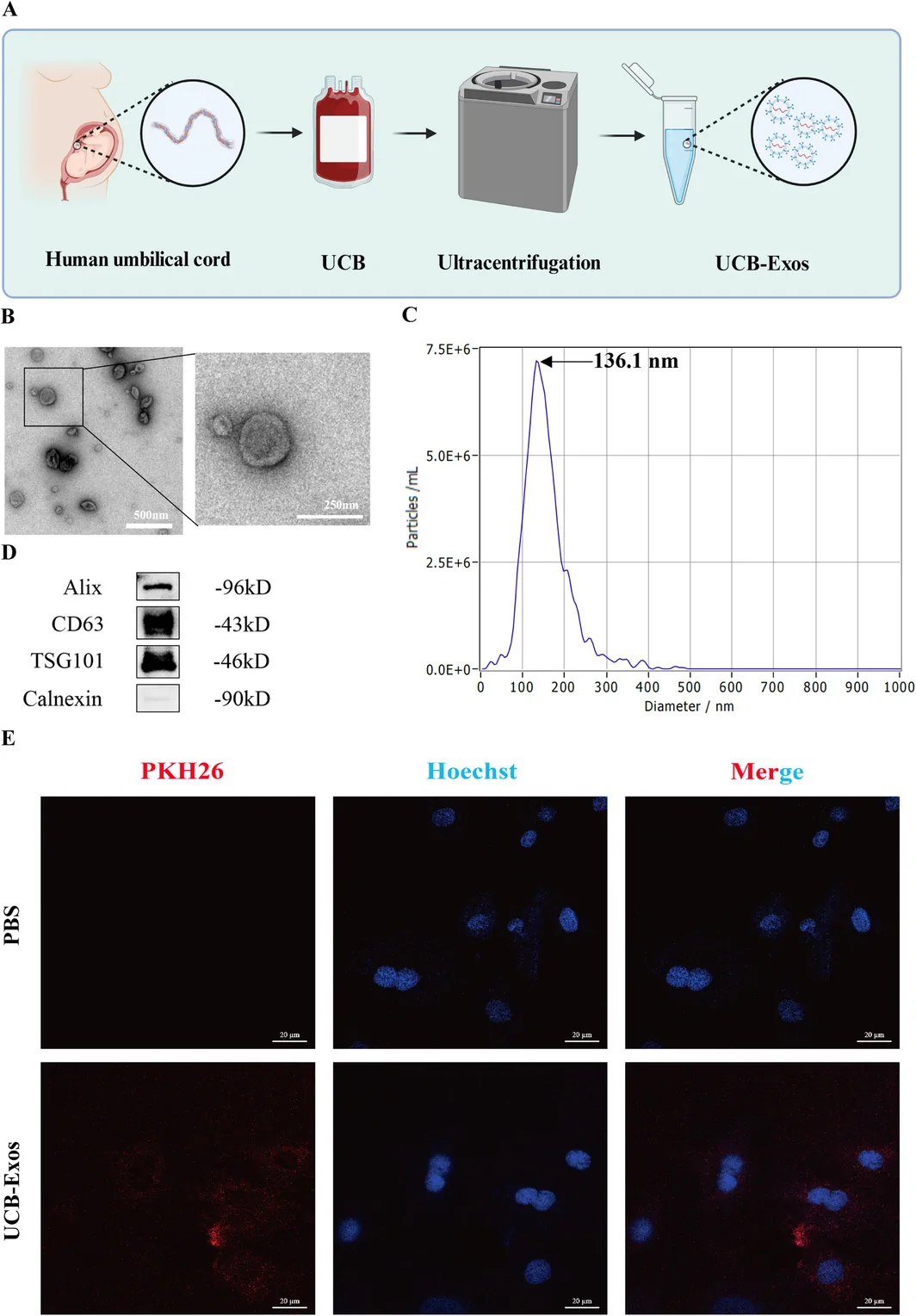
Isolation and identification of UCB‐Exos. (A) Flowchart of the isolation of UCB‐Exos. (Created **with** BioRender.com) (B) Morphology of UCB‐Exos observed using transmission electron microscopy. Scale bar = 500 nm. (C) Particle size distribution of UCB‐Exos measured using NTA analysis. (D) The surface markers of UCB‐Exos (CD63, ALIX, TSG101) were detected by Western blot. (E) The cellular internalisation of UCB‐Exos by BMSCs. UCB‐Exos were labelled with PKH‐26 (red), and the nuclei of BMSCs were stained with Hoechst 33,342 (blue). Scale bar = 20 μm.

### 3.2 Characterisation of GelMA/AlgMA‐Exos Composite Hydrogel

GA‐Exos composite hydrogel was successfully synthesised. Before photocrosslinking, it exhibited fluidity and injectability; after solidification, scanning electron microscopy (SEM) images showed a loose and porous structure with an average pore size of 12.32 ± 3.72 μm (Figure 3A–D, Figure S1). Fourier transform infrared (FTIR) spectroscopy confirmed the successful crosslinking of AlgMA and GelMA. Changes in characteristic absorption peaks indicated structural modification through covalent bonds, verifying the effectiveness of material synthesis (Figure 3E).

**FIGURE 3:**
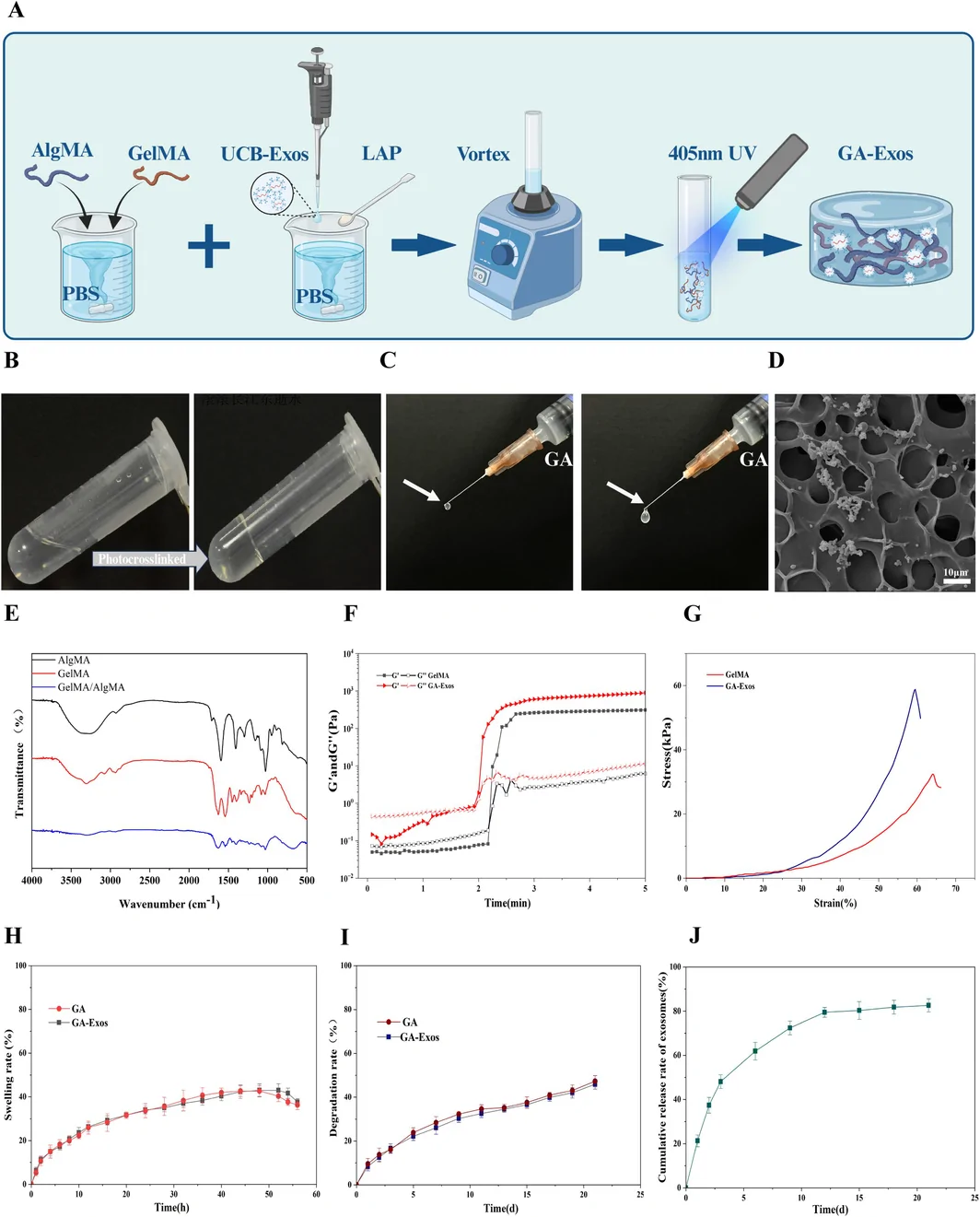
Characterisation of GA composite hydrogels. (A) Schematic diagram of GA‐Exos synthesis (Created with https://biorender.com). (B) Macroscopic morphology of GA composite hydrogel after photocrosslinking and solidification. (C) Injectability test of GA composite hydrogel. (D) Microstructure of GA composite hydrogel observed by scanning electron microscopy (SEM) (Scale bar: 10 μm). (E) Fourier transform infrared (FTIR) spectroscopy analysis: Spectra of GelMA, AlgMA, and GelMA/AlgMA in the range of 400–4000 cm^−1^. (F) Rheological property test of GelMA and GA‐Exos composite hydrogels. (G) Compressive stress–strain curves of GelMA and GA‐Exos composite hydrogels. (H) Swelling kinetic curves of GA and GA‐Exos composite hydrogels. (I) In vitro degradation curves of GA and GA‐Exos composite hydrogels. (J) Cumulative release rate of UCB‐Exos from GA‐Exos composite hydrogel on Days 1, 2, 3, 6, 9, 12, 15, 18, and 21.

Rheological analysis revealed that the composite hydrogel was in a viscoelastic liquid state before light irradiation (G″ > G′). After photocuring, the storage modulus (G′) of GA‐Exos was much higher than that of pure GelMA, with superior gelation kinetics, forming a stable elastic‐dominant network (Figure 3F). In compression tests, GA‐Exos achieved a peak compressive stress of 58.78 kPa at 60% strain, which was significantly higher than that of GelMA (32.44 kPa at 65% strain). These results demonstrated that GA‐Exos had better stiffness and compression resistance, making it suitable for the oral mechanical microenvironment (Figure 3G).

Regarding swelling and degradation properties, no significant differences were observed between GA and GA‐Exos: GA reached a swelling ratio of 42.66% at 44 h, while GA‐Exos reached 43% at 48 h, and both tended to stabilise after 48 h (*p* > 0.05). The in vitro degradation rates at 21 days were 47.3% and 45.9%, respectively (*p* > 0.05), indicating that exosome loading did not significantly alter the water absorption and degradation kinetics of the hydrogel (Figure 3H,I). Exosome sustained‐release tests showed that GA‐Exos achieved a cumulative release rate of 48.1% on day 3% and 82.6% on day 21 (Figure 3J).

### 3.3 Biocompatibility of GA and GA‐Exos Hydrogels

Calcein/PI staining and CCK‐8 assay showed that neither GA nor GA‐Exos hydrogel exhibited significant cytotoxicity. Co‐culture with GA‐Exos significantly enhanced the proliferation of BMSCs at 48 h, whereas GA exerted no significant effect on BMSC proliferation (Figure 4A,B).

**FIGURE 4:**
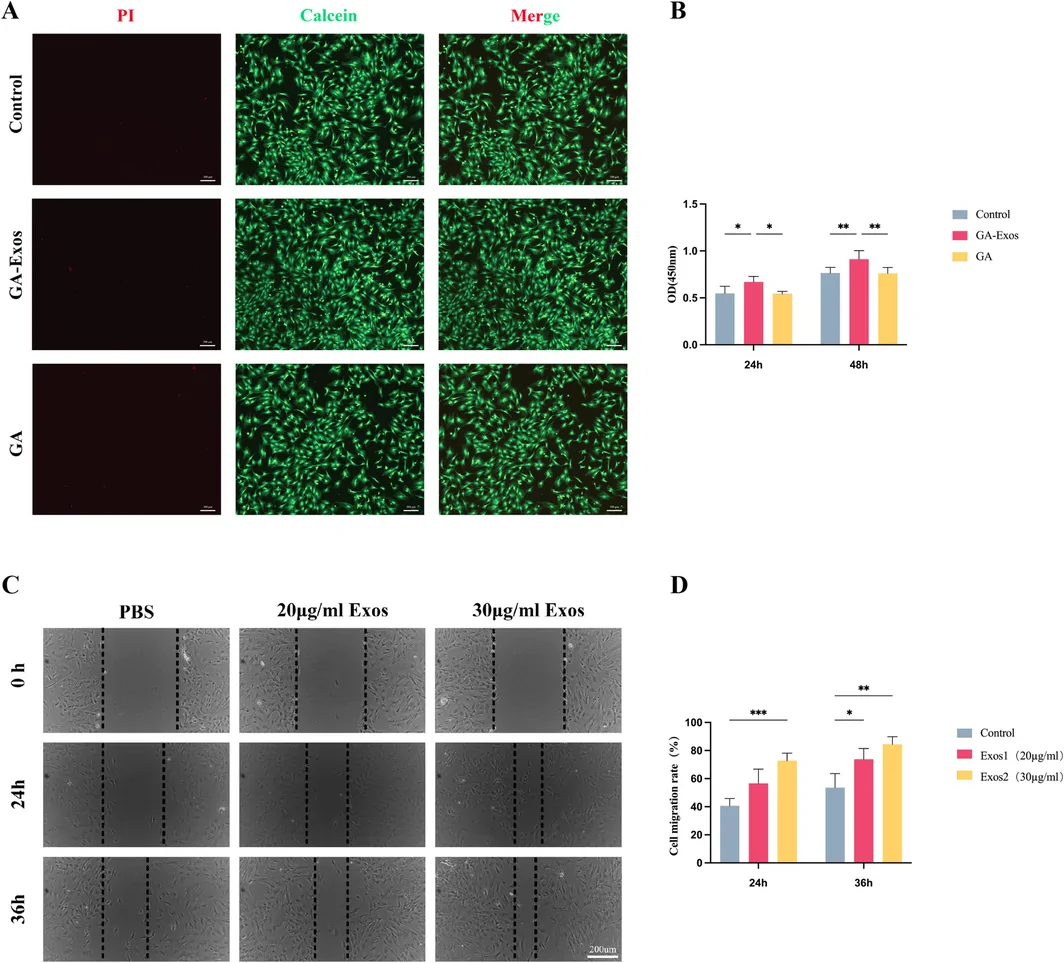
The biocompatibility of the GA composite hydrogel and UCB‐Exos promoted the proliferation and migration of BMSCs. (A) A Live/dead staining of BMSCs on the hydrogel after 2 days of incubation. Scale bar = 200 μm. (B) The effect of UCB‐Exos on the proliferation of BMSCs was detected by the CCK‐8 assay. (C) The effect of UCB‐Exos on the migration of BMSCs was detected by the cell scratch assay. Scale bar = 200 μm. (D) Quantitative analysis of migration rates. *p < 0.05; **p < 0.01; ***p < 0.001; ****p < 0.0001; ns, no significance.

### 3.4 Promotion of Cell Migration by UCB‐Exos

Cell Scratch Assay results demonstrated that UCB‐Exos at concentrations of 20 μg/mL and 30 μg/mL significantly enhanced BMSC migration after 24 h and 36 h of co‐culture, with the 30 μg/mL group showing a more pronounced promotional effect (Figure 4C,D).

### 3.5 Promotion of Osteogenic Differentiation by UCB‐Exos in Vitro

Co‐culture experiments of GA‐Exos composite hydrogel with BMSCs confirmed its osteogenic activity: ALP staining showed that the stained area in the GA‐Exos group was significantly larger than that in the control group on days 3 and 7 (Figure 5A); ARS staining revealed the formation of calcium nodules in all groups on days 14 and 21, with the GA‐Exos group exhibiting the strongest osteogenic induction capacity, while no significant difference was observed between the GA group and the control group (Figure 5B); both qRT‐PCR (day 7) and Western blotting results demonstrated that the expression levels of osteogenic‐related genes (ALP, RUNX2, OPN) and proteins were significantly upregulated in the GA‐Exos group (Figure 5C–I).

**FIGURE 5:**
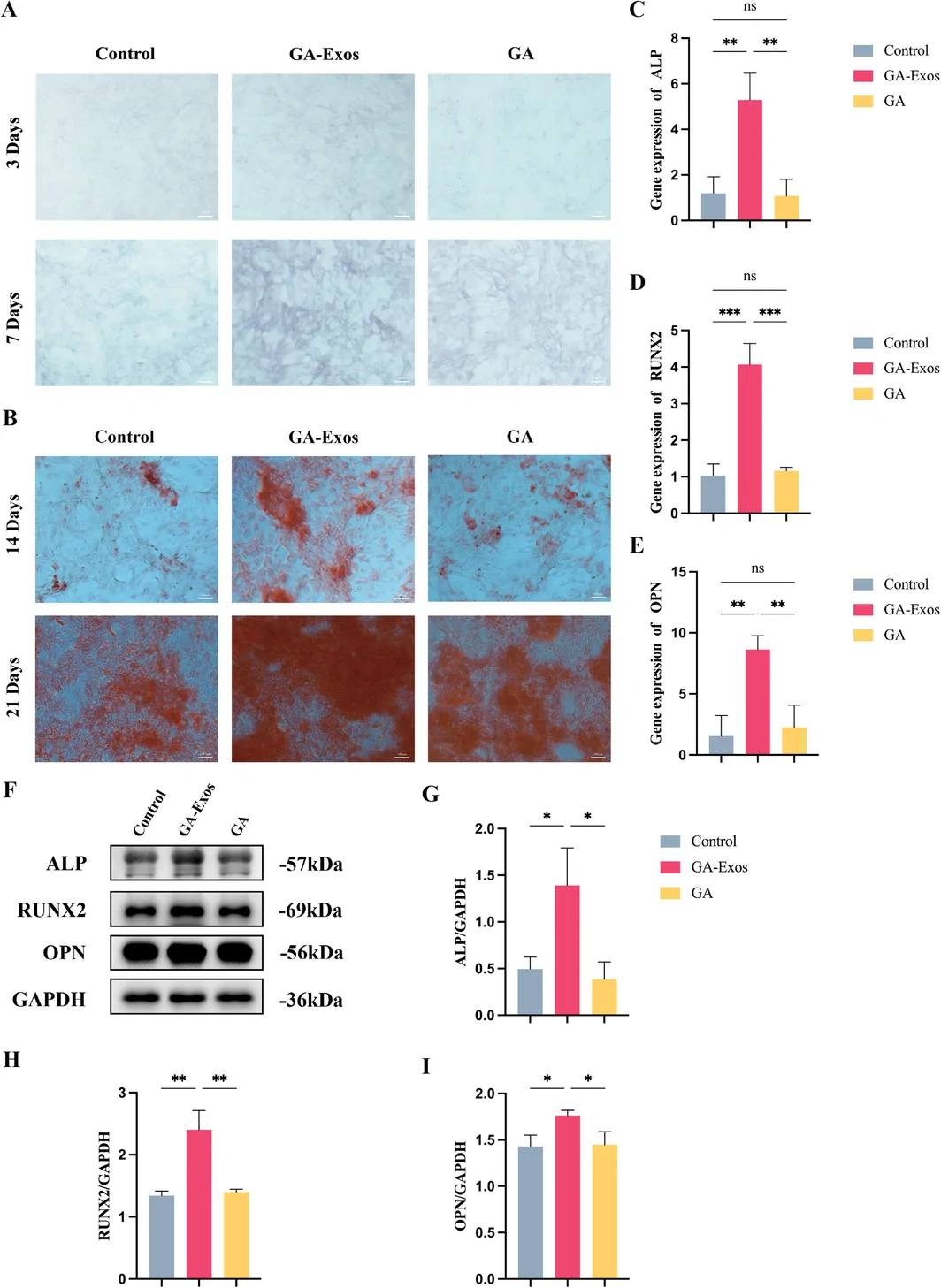
GA‐Exos promoted osteogenic differentiation of BMSCs. (A) ALP expression was assessed at days 3 and 7 (using ALP staining). Scale bar = 100 μm. (B) Calcium nodule formation (using ARS staining) was assessed at days 14 and 21. Scale bar = 100 μm. (C–E) Expression of osteogenic genes (RUNX2, ALP, OPN) in BMSCs for 7 days after induction. (F) Expression of osteogenic proteins (RUNX2, ALP, OPN) in BMSCs for 7 days after induction. (G–I) Quantitative analysis of grey values in Western blots. *p < 0.05; **p < 0.01; ***p < 0.001; ****p < 0.0001; ns, no significance.

### 3.6 UCB‐Exos Promote Periodontal Inflammatory Defects of Bone Regeneration in Vivo

A rat model of periodontitis was established in SD rats via silk suture ligation combined with local LPS injection. After 4 weeks of induction, clinical symptoms including gingival swelling, bleeding, and tooth mobility were observed, and CBCT scans revealed radiolucent areas around the maxillary first molars, confirming the successful establishment of the periodontitis model (Figure 6A,B). Following debridement, rats were treated with PBS, GA, or GA‐Exos, respectively. The results showed that at 4 weeks post‐treatment, gingival inflammation was significantly alleviated in the GA‐Exos group, with significantly downregulated expression of the inflammatory factors TNF‐α and IL‐1β (Figure 6C,D,F,G); CBCT scans performed 2 weeks post‐treatment showed a significant increase in bone volume in the GA‐Exos group (Figure 6E); micro‐CT analysis after 4 weeks of treatment revealed that the GA‐Exos group exhibited significantly increased bone volume fraction (BV/TV), mean CT value, trabecular thickness (Tb. Th), and trabecular number (Tb. N), along with a significant decrease in alveolar bone crest‐cementoenamel junction distance (ABC‐CEJ) (Figure 7A–F); H&E staining showed reduced inflammatory cell infiltration, with newly formed bone and vascular tissues observed in the GA‐Exos group (Figure 7G); TRAP staining indicated that the number of TRAP‐positive osteoclasts was lower in the GA‐Exos group than in the control and GA groups (Figure 7H,I).

**FIGURE 6:**
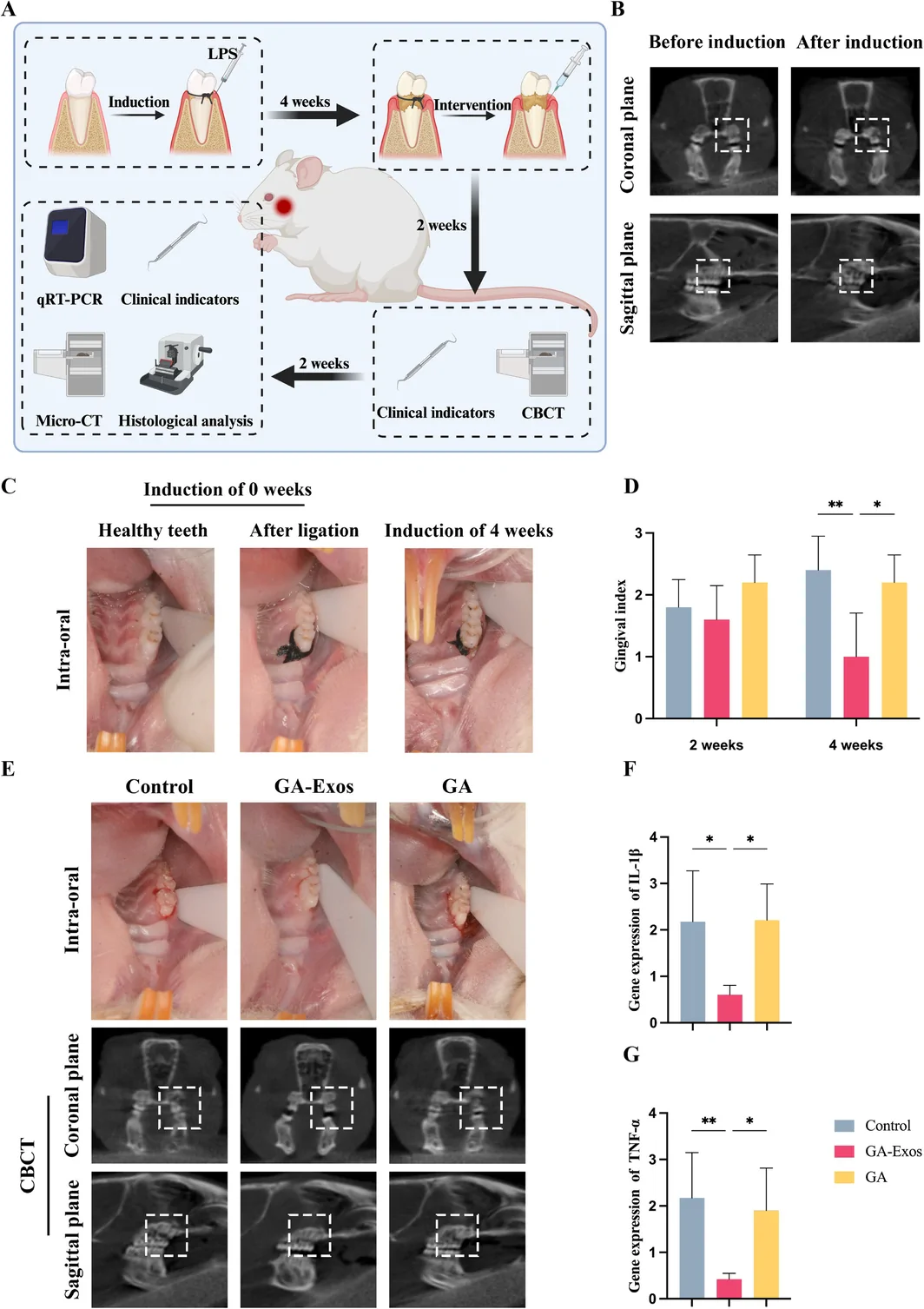
GA‐Exos promoted bone healing of inflammatory bone defects caused by periodontitis in rats. (A) Flow chart of in vivo experiments. (Created **with** BioRender.com) (B) The effect of periodontitis induction in the rat model was evaluated by CBCT at the fourth week after induction. (C) Intraoral conditions in rats during induction. (D) The gingival index of the rats was recorded at the second and fourth weeks of intervention. (E) In the second week of intervention, CBCT and intraoral photographs were used to evaluate the therapeutic effects of PBS, GA‐Exos and GA on periodontitis. In rats. (F, G) In the fourth week of intervention, the gingival tissues around the affected teeth were collected to analyse the gene expression of inflammatory factors (TNF‐α and IL‐1β) by qRT‐PCR. *p < 0.05; **p < 0.01; ***p < 0.001; ****p < 0.0001; ns, no significance.

**FIGURE 7:**
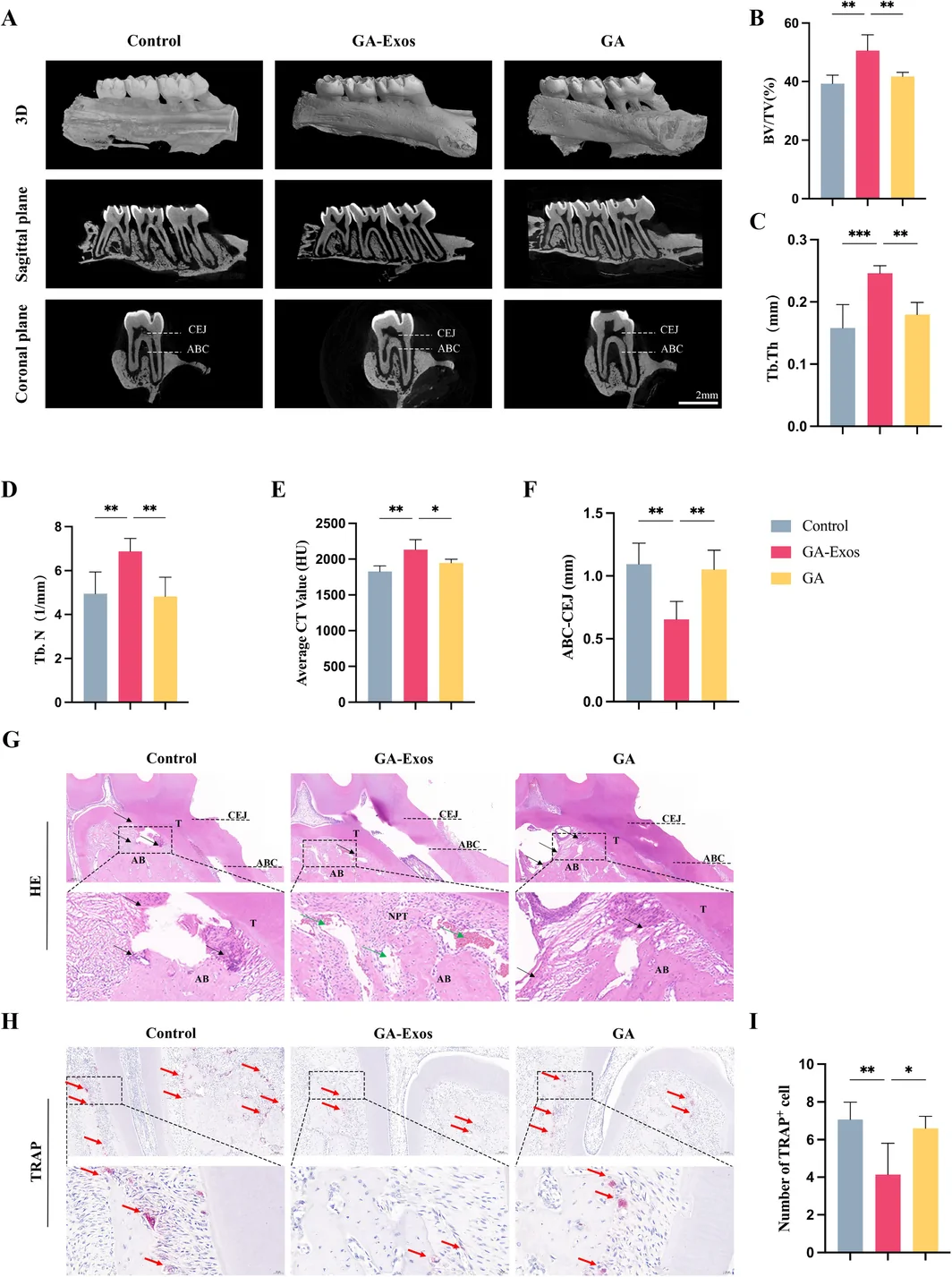
Micro‐CT and histology were used to analyse the effect of GA‐Exos in the treatment of periodontitis. (A) Representative micro‐CT images showing the alveolar bone status of each group at the fourth week (ABC: Alveolar bone crest; CEJ: Cementoenamel junction). (B–F) Quantitative micro‐CT assessment of BV/TV (%), Tb. Th (mm), Tb. N (1/mm), Average CT Value (HU), ABC‐CEJ (mm). (G) Representative images stained with H&E. New periodontal tissue and blood vessels (green arrows) were observed in the GA‐Exos group, and more inflammatory cells (black arrows) were observed in the Control and GA groups (NPT: New periodontal tissue; AB: Alveolar bone; T: Tooth tissue). Scale bar = 50 μm/200 μm. (H) The degree of osteoclast infiltration in representative TRAP‐stained sections of periodontal tissues (red arrow). The GA‐Exos group had fewer TRAP‐positive cells than the other two groups. Scale bar = 20 μm/100 μm. (I) Quantitative analysis of osteoclast number in the first molar region. *p < 0.05; **p < 0.01; ***p < 0.001; ****p < 0.0001; ns, no significance.

### 3.7 Identification of DEGs and Functional Analysis of These DEGs

mRNA sequencing results of cells from the Exos group and control group showed good sample correlation within and between groups (Figure S2A). A total of 1381 DEGs were identified with the criteria of |log2FoldChange| > 1 and *p* < 0.05, including 440 upregulated and 941 downregulated genes (Figure 8A, S2B). Bidirectional hierarchical clustering effectively distinguished the two groups (Figure 8B). KEGG and GO enrichment analyses revealed that DEGs were mainly enriched in the Toll‐like receptor signalling pathway, NF‐κB signalling pathway, and immune‐related pathways (Figure 8C,D, S3A‐B). GSEA confirmed that the Toll‐like receptor and NF‐κB signalling pathways were significantly downregulated (normalised enrichment score [NES] < −1, *p* < 0.001, false discovery rate [FDR] < 0.25) (Figure 8E,F, S4B). Additionally, the expression levels of LPS‐related receptors (LBP, CD14, TLR4) were significantly decreased (Figure 8G,H, S4A).

**FIGURE 8:**
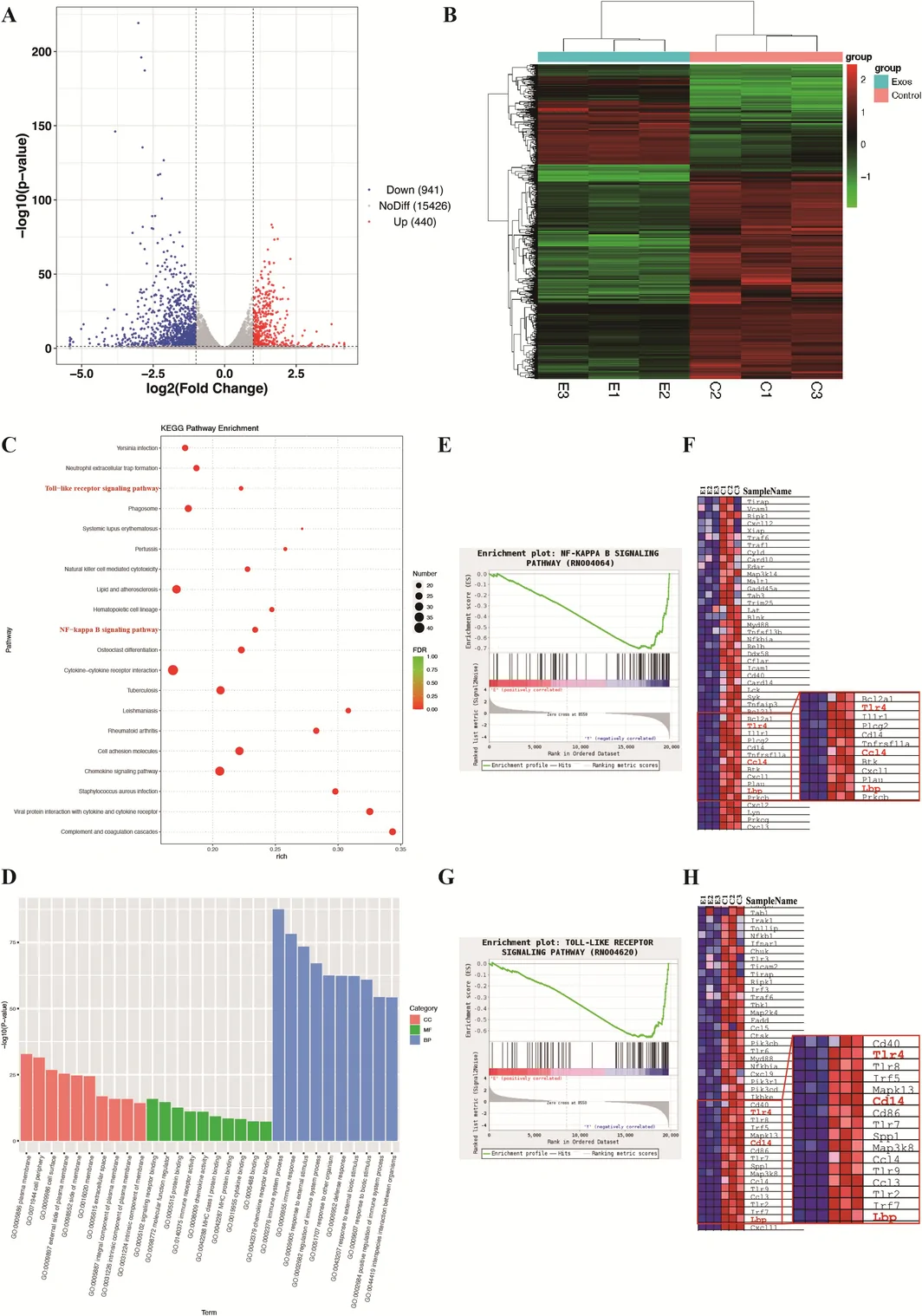
Identification of DEGs and functional analysis of these DEGs. (A) DEGs volcano plot, |log2FoldChange| > 1 and *p*‐value < 0.05. (B) DEGs heatmap. (C) KEGG pathway enrichment analysis of down‐regulated genes, *p*‐value < 0.05. (D) GO enrichment analysis of down‐regulated genes, *p*‐value < 0.05. (E) GSEA enrichment analysis of the NF‐κB signalling pathway. (F) Heat map of NF‐κB signalling pathway genes (the complete heat map is shown in Figure S4B). (G) GSEA enrichment analysis of the Toll‐like receptor signalling pathway. (H) Heat map of Toll‐like receptor signalling pathway genes (the complete heat map is shown in Figure S4A).

### 3.8 UCB‐Exos Promote Osteogenic Differentiation by Downregulating the TLR4/NF‐κB Signalling Pathway

To validate the aforementioned mRNA sequencing results at the protein level, we further designed in vitro mechanistic experiments. First, qRT‐PCR verified that UCB‐Exos significantly downregulated the mRNA expression of LBP, CD14, and TLR4 (Figure 9A–D). More importantly, we performed Western blot analysis to assess the core proteins of the identified TLR4/NF‐κB pathway. WB results demonstrated that LPS could upregulate the expression of TLR4 and p‐P65, while UCB‐Exos reversed this effect (Figure 9E–G). Alizarin Red S (ARS) staining confirmed that LPS inhibited calcium nodule formation, whereas UCB‐Exos promoted osteogenic differentiation under inflammatory conditions (Figure 9H). Collectively, these results indicate that UCB‐Exos exert osteogenic‐promoting effects by downregulating the TLR4/NF‐κB signalling pathway.

**FIGURE 9:**
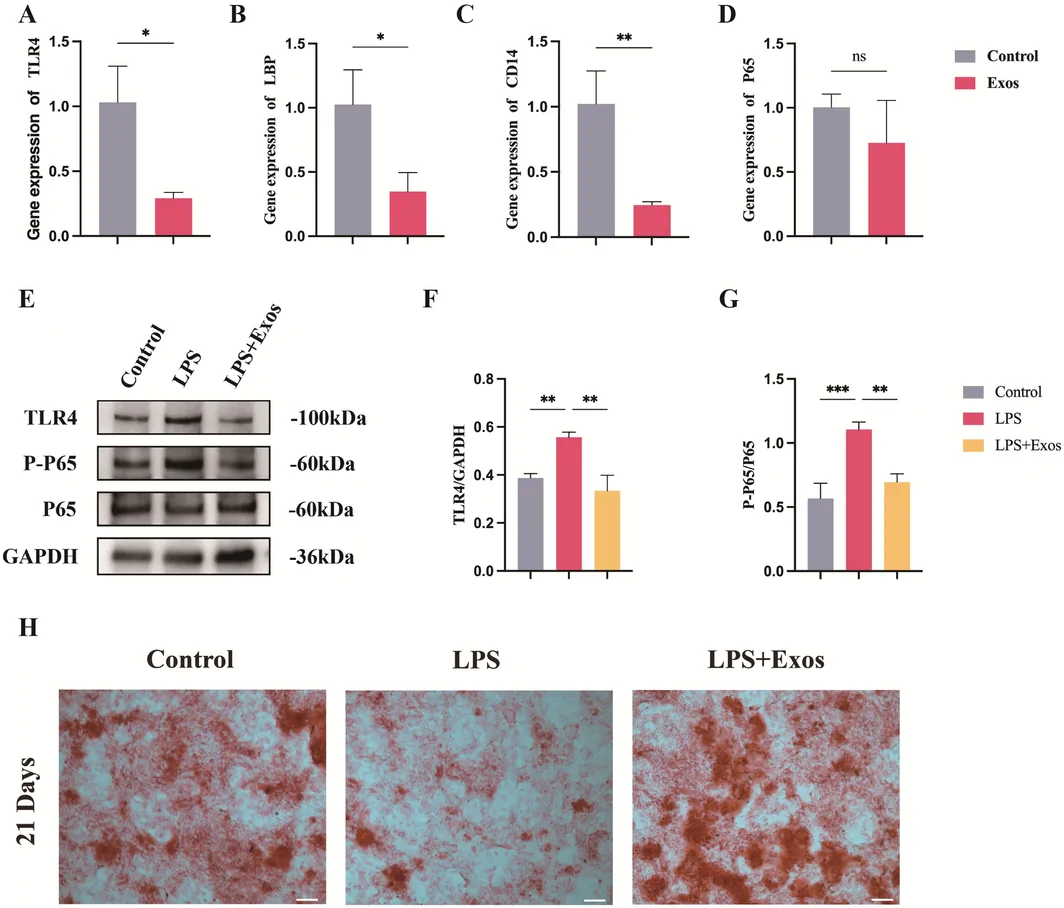
Validation of mRNA sequencing findings: UCB‐Exos promoted osteogenic differentiation by inhibiting LPS‐induced activation of the TLR4/NF‐κB pathway. (A–D) Gene expression of TLR4, LBP, CD14, and P65 after 7 days of co‐culture. (E) Protein expression of TLR4, P65 and P‐P65 in the Control group, LPS group and LPS + Exos group. (F, G) Quantitative analysis of grey values in Western blots. (H) Calcium nodule formation (using ARS staining) was assessed on day 21 in the Control group, LPS group and LPS + Exos group. Scale bar = 100 μm. *p < 0.05; **p < 0.01; ***p < 0.001; ****p < 0.0001; ns, no significance.

## 4 Discussion

The core pathological hallmarks of periodontitis include inflammatory cell infiltration and progressive bone resorption. The key to treatment lies in simultaneously achieving anti‐inflammation and repairing inflammatory bone defects, which remains a major challenge in the field of bone regeneration. Previous studies have demonstrated that human umbilical cord‐derived exosomes exhibit both anti‐inflammatory and osteogenic properties [21, 28, 29, 30], thereby providing a novel therapeutic approach for periodontitis. In this study, UCB‐Exos were loaded into a GelMA/AlgMA (GA) composite hydrogel, and the efficacy of this delivery system in repairing periodontal inflammatory bone defects was verified through in vitro and in vivo experiments.

As a cell‐free therapeutic strategy, exosomes, which are often categorised within the broader class of small extracellular vesicles (sEVs), have attracted considerable attention in the field of bone regeneration [31]. Derived from human umbilical cord blood, a medical waste‐derived resource, UCB‐Exos offer advantages such as easy accessibility, minimal ethical controversy, and low immunogenicity [20, 32]. In this study, UCB‐Exos meeting the physical criteria for sEVs set by MISEV2023 [27] were efficiently internalised by BMSCs and promoted cell proliferation, migration, and osteogenic differentiation. These findings are consistent with the conclusions reported by Ye et al. [20] and Hu et al. [33], laying a solid foundation for subsequent in vivo experiments.

The therapeutic efficacy of exosomes is highly dependent on delivery systems. Hydrogel‐mediated sustained‐release strategies can address the bottleneck of rapid in vivo clearance of exosomes [34, 35]. GelMA has emerged as an ideal carrier due to its excellent biocompatibility, but it suffers from weak mechanical properties [36, 37]. In this study, the introduction of AlgMA to construct a GA composite hydrogel significantly enhanced the mechanical stability of the material. Moreover, live/dead cell staining and CCK‐8 assays confirmed its superior biocompatibility, which is consistent with the research by Wei et al. [38]. More importantly, the GA hydrogel enabled the stable sustained release of UCB‐Exos. Together with studies by Lu et al. [39] and Xia et al. [40], our results confirm the application potential of composite hydrogels in long‐acting drug delivery.

Dysregulation of host immune inflammation is the core mechanism underlying tissue destruction in periodontitis [41]. Imbalance in macrophage M1/M2 phenotypes and excessive secretion of pro‐inflammatory cytokines such as TNF‐α and IL‐1β exacerbate tissue damage [12, 42]. In this study, after 4 weeks of GA‐Exos treatment, the expression levels of TNF‐α and IL‐1β in gingival tissues were significantly downregulated, and H&E staining showed reduced inflammatory cell infiltration, indicating the anti‐inflammatory effect of UCB‐Exos. Because TNF‐α and IL‐1β represent canonical functional products of M1‐polarised macrophages, their marked inhibition strongly indicates the resolution of M1‐driven local tissue hyper‐inflammation, consistent with previous findings [12, 41, 42, 43, 44]. These findings are consistent with the conclusions reported by Fan et al. [43] and Liu et al. [44]. Fan et al. [43] confirmed that exosomes exert anti‐inflammatory effects by regulating the TLR4/NF‐κB pathway, which aligns with the results of our GSEA enrichment analysis.

In in vivo experiments, CBCT and micro‐CT results demonstrated that GA‐Exos effectively repaired periodontal inflammatory bone defects, consistent with previous studies by Zhang et al. [45], NaKao et al. [46], Shen et al. [47], and Chen et al. [48]. TRAP staining confirmed that GA‐Exos reduced the number of osteoclasts, a finding closely related to the core mechanism of periodontitis‐induced bone defects: Dysregulation of the osteoblast–osteoclast balance [49]. The sustained release of UCB‐Exos from the hydrogel promotes regeneration via both direct cellular uptake (accelerating BMSC osteogenesis and inhibiting osteoclastogenesis) and indirect paracrine modulation (alleviating local inflammatory cytokines to reshape a favourable regenerative microenvironment).

mRNA sequencing in this study revealed the molecular mechanism underlying the osteogenic‐promoting effect of UCB‐Exos: A total of 1381 differentially expressed genes (DEGs) were identified, among which the TLR4/NF‐κB pathway and LPS‐related receptors (LBP, CD14, TLR4) were significantly downregulated (validated by qRT‐PCR). Previous studies have confirmed that TLR4‐mediated inflammatory responses regulate bone regeneration [50, 51, 52]. Yu et al. [51] found that upregulation of TLR4 inhibits the osteogenic differentiation of BMSCs, and this study is the first to observe that UCB‐Exos can significantly suppress LPS‐induced TLR4 expression. The TLR4 signalling pathway can activate NF‐κB, a key transcription factor in inflammatory responses that participates in the regulation of bone homeostasis [53], and its activation is closely associated with p65 phosphorylation [54]. Further mechanistic studies showed that UCB‐Exos inhibited LPS‐mediated p65 phosphorylation (with no difference in total p65 expression) and reversed the inhibitory effect of LPS on osteogenic differentiation (confirmed by ARS staining). These results suggest that UCB‐Exos exert osteogenic‐promoting effects by downregulating the TLR4/NF‐κB pathway, a mechanism that may be related to miRNAs delivered by UCB‐Exos and requires further investigation.

This study has several limitations: First, the rat model cannot fully recapitulate the pathological complexity of human periodontitis, and its translational potential requires further validation using more clinically relevant models; second, the donor heterogeneity of UCB‐Exos and the standardisation of isolation and production remain unresolved, and the key active components need to be clarified; third, while our current study demonstrated that UCB‐Exos downregulate the TLR4/NF‐κB pathway.

Alongside restoring osteogenesis, further loss‐of‐function or gain‐of‐function validation using specific inhibitors or gene manipulation will be required in future work to dissect the precise upstream microRNA/protein interactions. Future interdisciplinary collaborations to address these issues are expected to further enhance the efficacy and clinical translation potential of the GA‐Exos therapeutic strategy.

## 5 Conclusions

In conclusion, this study confirms that the GA composite hydrogel loaded with UCB‐Exos can promote bone tissue regeneration in periodontitis. The developed GA composite hydrogel exhibits injectability, sustained‐release properties, and excellent biocompatibility. In in vitro experiments, GA‐Exos enhanced the proliferation and osteogenic differentiation of BMSCs; in in vivo experiments, GA‐Exos effectively promoted maxillary bone regeneration while inhibiting inflammation and osteoclast differentiation. The underlying mechanism is that UCB‐Exos reverse the bacterial LPS‐activated TLR4/NF‐κB signalling pathway. This work provides a foundation for the development of novel therapeutic strategies for inflammatory bone defects caused by periodontitis.

## Author Contributions

**Dongao Zhu:** writing – original draft, investigation. **Mingkun Liu:** writing – original draft, investigation. **Yue Fan:** data curation, formal analysis. **Zuojun Shen:** conceptualization, validation, supervision. **Zhihong Zhang:** conceptualization, validation, supervision. **Honghong Liu:** conceptualization, supervision, funding acquisition. **Lv Lang:** data curation, formal analysis. **Xin Luo:** data curation, formal analysis.

## Funding

This work was supported by the Scientific Research Project of Colleges and Universities in Anhui Province (2023AH053394).

## Ethics Statement

All animal experimental protocols were approved by the Experimental Animal Ethics Committee of the First Affiliated Hospital of the University of Science and Technology of China.

## Conflicts of Interest

The authors declare no conflicts of interest.

## Supporting information


**Figure S1:** Pore size distribution of nGA composite hydrogels.
**Figure S2:** Correlation analysis of sequenced samples and MA map of DEGs. (A) Correlation analysis of sequenced samples. (Pearson's correlation coefficient represents the correlation of expression levels of genes between samples, and a correlation coefficient closer to 1 indicates a higher similarity of expression patterns between samples). (B) MA map of DEGs.
**Figure S3:** GO and KEGG enrichment analysis. (A) GO enrichment analysis bubble plot. (B) Bar graph of KEGG Pathway enrichment results.
**Figure S4:** Heat map of gene expression in Toll‐like receptor signalling pathway and NF‐κB signalling pathway. (A) Toll‐like receptor signalling pathway. (B) NF‐κB signalling pathway. (Red indicates upregulation and blue indicates downregulation).

## Data Availability

The data that support the findings of this study are available from the corresponding author upon reasonable request.
