# Supplementary material for: Injectable Hydrogel Loaded With Umbilical Cord Blood‐Derived Exosomes Promotes Periodontal Bone Regeneration via the TLR4/NF‐κB Signalling Pathway
Source: J Cell Mol Med. 2026 Sep 27;30(19):e71347. doi: 10.1111/jcmm.71347 (PMC13616824; doi:10.1111/jcmm.71347)
Supplement: Supplementary file 1 [file JCMM-30-e71347-s001.docx]

**Injectable Hydrogel Loaded with Umbilical Cord Blood-Derived Exosomes Promotes Periodontal Bone Regeneration via the TLR4/NF-κB Signaling Pathway**

**Dongao Zhu ^a, b, 1^, Mingkun Liu^a, b, 1^, Yue Fan^a, b^, Lv Lang ^a, b^, Xin Luo ^a, b^， Zhihong Zhang ^a, b*^, Zuojun Shen ^c *^, Honghong Liu ^a, b *^**

a Department of Stomatology, The First Affiliated Hospital of USTC, Division of Life Sciences and Medicine, University of Science and Technology of China, Hefei 230001, China

b **College of Stomatology, Bengbu Medical University, Anhui Bengbu 233000, China)**

c Department of Laboratory Medicine, The First Affiliated Hospital of USTC, Division of Life Sciences and Medicine, University of Science and Technology of China, Hefei 230001, China

1 Dongao Zhu and Mingkun Liu contributed equally to this manuscript.

* Correspondence: Honghong Liu ([66302784@qq.com](mailto:66302784@qq.com)), Zuojun Shen ([zuojunshen@ustc.edu.cn](mailto:zuojunshen@ustc.edu.cn)), Zhihong Zhang ([zzhzqr@126.com](mailto:luoxinkq@163.com)).

E-mail: Dongao Zhu ([zdahhhh@126.com](mailto:zdahhhh@126.com)), Mingkun Liu ([1019365327@qq.com](mailto:1019365327@qq.com)), Yue Fan ([690517413@qq.com](mailto:690517413@qq.com)), Lv Lang([1124173470@qq.com](mailto:1124173470@qq.com)), Xin Luo ([luoxinkq@163.com](mailto:luoxinkq@163.com))

**Figure S1.Pore size distribution of nGA composite hydrogels.**

**Figure S2. Correlation analysis of sequenced samples and MA map of DEGs.** (A) Correlation analysis of sequenced samples. (Pearson's correlation coefficient represents the correlation of expression levels of genes between samples, and a correlation coefficient closer to 1 indicates a higher similarity of expression patterns between samples.) (B)	MA map of DEGs.

**Figure S3. GO and KEGG enrichment analysis.** (A) GO enrichment analysis bubble plot. (B)	Bar graph of KEGG Pathway enrichment results.

**Figure S4.** **Heat map of gene expression in Toll-like receptor signaling pathway and NF-κB signaling pathway.** (A) Toll-like receptor signaling pathway. (B) NF-κB signaling pathway. (Red indicates upregulation and blue indicates downregulation.)
